# Obstetrical and perinatal outcomes of women with preeclampsia at Woldia Comprehensive Specialized Hospital, Northeast Ethiopia

**DOI:** 10.3389/fmed.2024.1326333

**Published:** 2024-09-18

**Authors:** Henok Kumsa, Desalew Mergiyaw

**Affiliations:** ^1^Department of Midwifery, College of Health Sciences, Woldia University, Woldia, Ethiopia; ^2^Godere Mission Health Center, Gambela, Ethiopia

**Keywords:** preeclampsia, adverse obstetrical outcomes, adverse perinatal outcomes, adverse obstetrical and perinatal outcomes, Woldia

## Abstract

**Background:**

Preeclampsia is a multisystem disorder that affects pregnant women. Preeclampsia and its complications are the leading causes of maternal and perinatal morbidity and mortality in developing countries. Studies conducted in Ethiopia have primarily concentrated on preeclampsia’s trends and prevalence rather than its obstetrical and perinatal consequences. Thus, this study aimed to determine the risk of adverse obstetric and perinatal outcomes among women with preeclampsia at Woldia Comprehensive Specialized Hospital, Northeast Ethiopia.

**Methods:**

A retrospective cohort study was conducted among 140 preeclamptic women and 280 normotensive women who gave birth at Woldia Comprehensive Specialized Hospital between 30 December 2020 and 29 December 2022. Maternal records were retrieved using data-extraction tools. The data were entered into EpiData version 4.6.0.6 and analyzed using SPSS version 26. Binary and multivariable logistic regression models were used to test the associations between independent and outcome variables. The adjusted odds ratio (OR) with a 95% confidence interval (CI) and *p*-values <0.05 were used to measure the strength of the association and declare the level of statistical significance.

**Results:**

The odds of at least one adverse obstetric outcome among preeclamptic women were 2.25 times higher than those among normotensive women [AOR: 2.25, 95% CI: (1.06, 4.77)]. In addition, babies born to preeclamptic women were at a higher risk of perinatal death [AOR: 2.90, 95% CI: (1.10, 8.17)], low birth weight [AOR: 3.11, 95% CI: (1.43, 6.7)], birth asphyxia [AOR: 2.53, 95% CI: (1.15, 5.5)], and preterm birth [AOR: 2.21, 95% CI: (1.02, 4.8)] than babies born to normotensive women.

**Conclusion:**

More adverse obstetric and perinatal outcomes were observed in women with preeclampsia than those in normotensive women. This study highlights the significantly elevated level of at least one adverse obstetric outcome associated with preeclampsia, low hemoglobin level, and rural residents. Moreover, perinatal death, low birth weight, asphyxia, and preterm birth were significantly associated with preeclampsia.

## Background

Preeclampsia is a hypertensive disorder that typically occurs in pregnancy after 20 weeks of gestation. It is characterized by the new onset of high blood pressure (hypertension) and the presence of protein in the urine (proteinuria). However, it is important to note that preeclampsia can also manifest without significant proteinuria, and in some cases, it may present with other signs of end-organ dysfunction, such as impaired liver function, kidney dysfunction, blood clotting abnormalities, or neurological symptoms (1, 2).

The exact etiology of preeclampsia is not fully understood. However, this pregnancy-related condition is linked to factors such as placental dysfunction, impaired vascular remodeling, and endothelial dysfunction. These factors lead to reduced blood flow and inflammation, which contribute to the characteristic symptoms of preeclampsia (3). Its hallmark features include high blood pressure (BP) and widespread end-organ injury or damage. However, the clinical presentation can be highly variable and perplexing: elevated BP may precede or follow proteinuria, other symptoms may be masked or absent, and its onset can be gradual, abrupt, or even present in the postpartum period (4).

The incidence of preeclampsia varies across regions; it affects 3–5% of pregnant women worldwide. However, evidence suggests that the prevalence of preeclampsia is higher in low-income countries, with reported rates ranging from 1.8 to 16.7% (5). It is one of the top five leading causes of maternal and perinatal morbidity and mortality worldwide, especially at an early onset (6, 7). Globally, approximately 76,000 mothers and 500,000 babies die annually because of preeclampsia (8). Likewise, in sub-Saharan African countries, hypertensive disorder of pregnancy was the second most common cause of maternal death, accounting for approximately 22.1% (95% CI: 19.9–24.2%) of maternal deaths (9). The overall adverse obstetric and perinatal outcomes in preeclampsia are worse and higher in women with severe preeclampsia and eclampsia (6).

In Ethiopia, the prevalence of preeclampsia ranges from 2.23 to 12.4% (10–13), contributing to 16% of direct maternal mortality (14). A hospital-based study conducted in the Amhara region reported that preeclampsia and eclampsia accounted for 35% of maternal deaths (15). A similar study conducted in Ethiopia revealed that 33.5% of unfavorable maternal outcomes were observed among women with preeclampsia (16). Additionally, studies conducted in different settings in Ethiopia showed that more than half of the perinatal outcomes in preeclamptic women were complicated (16, 17).

Reducing maternal and neonatal mortality is a significant global health priority, prompting countries to adopt diverse strategies in their pursuit of the Sustainable Development Goals. These goals aim to eliminate preventable deaths among newborns, achieve a global neonatal mortality rate of 12:1000 live births or less, and reduce the global maternal mortality ratio to less than 70/100,000 births by 2030 (18).

Numerous studies, both in developed and developing countries, have been conducted to investigate the maternal and perinatal outcomes related to preeclampsia (19–22). However, there are only limited studies that focus on pregnancy outcomes specifically associated with this disease in Ethiopia, especially in Woldia comprehensive specialized hospital (WCSH). Therefore, this study aimed to determine the risk of adverse obstetric and perinatal outcomes among women with preeclampsia at Woldia comprehensive specialized hospital, Northeast Ethiopia.

## Methods

### Study area, design, and period

The study was conducted at WCSH, found in the Amhara region of northern Ethiopia. WCSH is located in Woldia town, which is approximately 365 km northeast of Bahir Dar and 520 km northeast of Addis Ababa. The hospital serves a population of approximately 3 million residents in the nearby regions and zones. According to the hospital’s annual service reports, it provides care for over 4,500 women who give birth each year. A retrospective cohort study was conducted from 1 April to 1 May 2023.

### Exposure ascertainments

We determined the exposure of interest based on the guidelines outlined in the Obstetrics Management Protocol for Hospitals in Ethiopia (2021) and the most recent recommendations from the International Society for the Study of Hypertension in Pregnancy (23). In this study, the main exposure variable was preeclampsia. It is defined as the occurrence of new-onset hypertension and proteinuria after 20 weeks of gestation in women who were previously normotensive. Hypertension is characterized by systolic blood pressure (BP) ≥ 140 mmHg and/or diastolic BP ≥ 90 mmHg on at least two occasions, with a time interval of 4 hours. Alternatively, in the absence of proteinuria, new-onset hypertension can be accompanied by thrombocytopenia, renal insufficiency, impaired liver function, pulmonary edema, or new-onset medication-resistant headache. Preeclampsia can be further classified into different types based on its severity, onset, and the presence of specific complications (24). Normotensive women in this study were defined as pregnant individuals whose blood pressure (BP) measurements were below 140/90 mmHg at 20 or more weeks of gestation and who did not develop preeclampsia or proteinuria during their pregnancy (25).

### Source and study population

The study included all mothers who gave birth at WCSH, located in Northeast Ethiopia, considered as the source population. The study population comprised all mothers with and without preeclampsia who gave birth at WCSH in Northeast Ethiopia from 30 December 2020 to 29 December 2022.

### Eligibility criteria and study variables

Medical records of pregnant mothers with and without preeclampsia who gave birth at WCSH were included in the study. However, mothers diagnosed with chronic hypertension, eclampsia, multiple pregnancies, or gestational diabetes mellitus were excluded from the study. In the study, adverse obstetrical and/or perinatal outcomes were considered the dependent variables, with maternal preeclampsia as the main exposure variable. Sociodemographic covariates included the mother’s age and place of residence. Additionally, obstetric characteristics and reproductive healthcare service utilization-related covariates were assessed, including parity, antenatal care follow-up, number of ANC visits, iron and folic acid supplementation, gestational age at delivery, onset of labor, mode of delivery, duration of labor, and hemoglobin level.

### Sample size determination

The sample size for the study was calculated using the double population proportion formula in Epi Info version 7.2.5. The calculation was based on a 95% confidence level, 80% power, and an assumed ratio of 1: 2 between women with preeclampsia and those without preeclampsia. In a study conducted in the Sidama region, Ethiopia, the proportion of cesarean deliveries in pregnant women with preeclampsia and without preeclampsia was 45.1 and 29.9%, respectively (17). After adding 10% of the missing charts, the final sample size was 420 (140 women with preeclampsia and 280 without non-preeclampsia).

### Sampling technique and procedures

First, all women who gave birth at the WCSH between 30 December 2020 and 29 December 2022 were identified from the delivery registration logbook, totaling 5,923. Among them, 253 had preeclampsia, while 5,593 did not. The study included mothers who met the eligibility criteria, and separate sampling frames were prepared for both groups using the delivery registration book. The medical record number was used as a frame, and a simple random sampling technique was employed to select a total of 420 records. This comprised 140 records from women with preeclampsia and 280 records from women without preeclampsia.

### Data collection instrument and procedures

The data were collected using a structured checklist adapted from previous studies with certain modifications. These included details on sociodemographic characteristics, obstetric and reproductive healthcare service utilization, and obstetric and perinatal outcomes. The data collection instrument was developed in the English language. To collect the data, three BSc midwives and one supervisor with 2 years of professional experience were recruited. The investigators reviewed the data daily to ensure their completeness.

### Data quality management

To ensure data quality, data collectors and supervisors underwent a one-day training session in line with the aim of the research, contents of the checklist, obstetric admission-discharge logbook, and issues related to confidentiality. A preliminary review was conducted on a 5% subset of a similar study population, which was not part of the actual sample. This review took place 1 week prior to data collection, and necessary corrections and modifications were made based on the findings from the preliminary review.

### Data processing and analysis

After the data were coded, they were entered into EpiData version 4.6.0.6 and then transferred to SPSS version 26 for further analysis. The model fitness was assessed using the Hosmer–Lemeshow goodness-of-fit test, while multicollinearity was checked using the variance inflation factor (VIF). A binary logistic regression model was employed to analyze the variables. Variables that had a *p*-value of <0.25 in the bivariate analysis were included in the multivariable logistic regression model. Adjusted odds ratios (ORs) with 95% confidence intervals (CI) and *p*-values of <0.05 were used to determine the strength of the associations and identify statistically significant results.

### Operational definitions

- Adverse obstetric outcomes in this study were defined as mothers who experienced at least one of the following complications: blood transfusion, antepartum hemorrhage, postpartum hemorrhage, pulmonary edema, or maternal death (26).
- Favorable (without adverse) perinatal outcomes: Newborns born to women with or without preeclampsia had no perinatal complications (25).
- Adverse perinatal outcomes: newborns born to women with and without preeclampsia had at least one complication (low birth weight, intrauterine growth restriction, preterm birth, low APGAR score at 5 min, perinatal asphyxia, or perinatal death) (21).

## Results

### Sociodemographic characteristics of the study participants

A total of 420 participant charts (140 preeclamptic women and 280 normotensive women) were reviewed. The mean maternal age was similar between women with preeclampsia and normotensive women, with both groups having a mean age of 27. The standard deviation (SD) for maternal age was ±6.00 for women with preeclampsia and ± 5.00 for normotensive women. The majority of women with and without preeclampsia were in the age range of 20–34 years old. Compared to normotensive women (15.7%), the majority of preeclamptic women (28.6%) were rural inhabitants (Table 1).

**Table 1:** Sociodemographic characteristics of the study participants at WCSH, Northeast Ethiopia, 2023.

Variables	Categories	Admission status of mothers		Total
		Women with preeclampsia	Normotensive women	
		*N* (%)	*N* (%)	*N* (%)
Age category	15–19	8 (5.7%)	14 (5.0%)	22 (5.24%)
	20–34	107 (76.4%)	227 (81.1%)	334 (79.52%)
	35–49	25 (17.9%)	39 (13.9%)	64 (15.24%)
Residence	Rural	40 (28.6%)	44 (15.7%)	84 (20.00%)
	Urban	100 (71.4%)	236 (84.3%)	336 (80.00%)

Obstetrics characteristics and reproductive healthcare services utilization of the participants.

### Obstetrics characteristics and reproductive health care services utilization of the participants

Most of the study participants, 129 (92.1%) and 260 (92.9%) of women with preeclampsia and normotensive women, had at least one ANC follow-up, respectively. Overall, 69 (49.3%) and 125 (44.6%) women with preeclampsia and normotensive, respectively, were nulliparous. Regarding the mode of delivery, 45 (32.1%) preeclamptic women gave birth through cesarean delivery compared to 61 (21.8%) without preeclampsia. Among women with preeclampsia, 41 (29.30%) were initiated by induction, compared to 15 (5.4%) without preeclampsia (Table 2).

**Table 2:** Obstetric characteristics and reproductive healthcare services utilization of the study participants at WCSH, Northeast Ethiopia, 2023.

Variables		Admission status of mothers		Totals
		Women with preeclampsia	Normotensive women	
		*N* (%)	*N* (%)	*N* (%)
ANC follow-up	Yes	129 (92.1)	260 (92.90)	389 (92.62)
	No	11 (7.9)	20 (7.1)	31 (7.38)
Number of ANC visits	Less than four	46 (35.7)	19 (7.3)	65 (15.48)
	Four and above	83 (64.3)	241 (92.7)	324 (77.14)
Iron folic acid supplementation	Yes	125 (89.3)	253 (90.4)	378 (90)
	No	15 (10.7)	27 (9.6)	42 (10)
Number of children	Null	69 (49.3)	125 (44.6)	194 (46.19)
	Primiparous	24 (17.1)	65 (23.2)	89 (21.19)
	Multiparous	47 (33.6)	90 (32.1)	137 (32.62)
Gestational age in weeks	<37 weeks	42 (30.0)	16 (5.7)	58 (13.81)
	≥ 37 weeks	98 (70.0)	264 (94.3)	362 (86.19)
Onset of delivery	Spontaneous	99 (70.7)	265 (94.6)	364 (86.67)
	Induction	41 (29.3)	15 (5.4)	56 (13.33)
Mode of delivery	SVD	84 (60.0)	207 (73.9)	291 (69.29)
	Instrumental [vacuum and forceps]	11 (7.9)	12 (4.3)	23 (5.48)
	Cesarean section	45 (32.1)	61 (21.8)	106 (25.24)
Duration of labor in hours	<12 h	90 (64.3)	126 (45.0)	216 (51.43)
	≥12 h	50 (35.7)	154 (55.0)	204 (4857)
Hemoglobin level in g/dl	<11 mg/dL	30 (21.4)	39 (13.9)	69 (16.43)
	≥11 mg/dL	110 (78.6)	241 (86.1)	351 (83.57)

### Magnitude of adverse obstetrical and perinatal outcomes

This study’s findings indicated that the prevalence of at least one adverse maternal outcome was higher among women with preeclampsia, with 31 (22.0%) of them experiencing such complications. In comparison, among normotensive women, the prevalence was lower, with 19 (6.80%) encountering adverse maternal outcomes. The proportion of blood transfusion, PPH, APH, and maternal death was higher in women with preeclampsia than in those without preeclampsia (9.3% vs. 2.9, 7.9% vs. 3.2, 5.7% vs. 2.1, and 0.7% vs. 0.0%, respectively). Moreover, the proportion of at least one adverse perinatal outcome among women with and without preeclampsia was 47.90 and 15.30%, respectively. The magnitudes of low birth weight, preterm birth, perinatal death, and perinatal asphyxia in the preeclampsia and normotensive groups were 31.4 and 7.1%, 30.0 and 6.1%, 16.40 and 2.40%, and 18.6 and 5.7%, respectively (Table 3).

**Table 3:** Magnitude of adverse obstetrical and perinatal outcomes of the study participant at WCSH, Northeast Ethiopia, 2023.

Adverse obstetrical outcome variables		Admission status of mothers		Total
		Women with preeclampsia	Normotensive women	
		*N* (%)	*N* (%)	*N* (%)
Adverse obstetrical outcomes	Yes	31 (22.10)	19 (6.80)	50 (11.90)
	No	109 (77.90)	261 (93.20)	370 (88.10)
Postpartum hemorrhage	Yes	11 (7.90)	9 (3.2)	20 (4.76)
	No	129 (92.10)	271 (96.8)	400 (95.24)
Antepartum hemorrhage	Yes	8 (5.70)	6 (2.10)	14 (3.33)
	No	132 (94.30)	274 (97.90)	406 (96.67)
Pulmonary edema	Yes	1 (0.70)	0 (0.00)	1 (0.24)
	No	139 (99.30)	280 (100.00)	419 (99.76)
Maternal death	Yes	1 (0.70)	0 (0.00)	1 (0.24)
	No	139 (99.30)	280 (100.00)	419 (99.76)
Blood transfusion	Yes	13 (9.30)	8 (2.9)	21 (5)
	No	127 (90.70)	272 (97.10)	399 (95)
Composite adverse perinatal outcomes	Yes	67 (47.90)	43 (15.40)	110 (26.19)
	No	73 (52.10)	237 (84.60)	310 (73.81)
Low birth weight	Yes	44 (31.40)	20 (7.10)	64 (15.24)
	No	96 (68.60)	260 (92.90)	356 (84.76)
Intrauterine growth restriction	Yes	13 (9.30)	2 (0.70)	15 (3.57)
	No	127 (90.70)	278 (99.30)	405 (96.43)
Preterm birth	Yes	42 (30.00)	17 (6.10)	59 (14.05)
	No	98 (70.00)	263 (93.90)	361 (85.95)
Perinatal asphyxia	Yes	26 (18.60)	16 (5.70)	42 (10)
	No	114 (81.40)	264 (94.30)	378 (90)
Perinatal death	Yes	23 (16.40)	8 (2.90)	31 (7.38)
	No	117 (83.60)	272 (97.10)	389 (92.62)
APGAR score at 5th min	<7	17 (14.40)	16 (5.80)	33 (7.86)
	≥7	101 (85.60)	259 (94.20)	360 (85.71)

### Association of maternal preeclampsia with adverse obstetrical and perinatal outcomes

Binary logistic regression analysis was conducted to assess the association between various sociodemographic factors, obstetric characteristics, and the utilization of reproductive health services with the occurrence of at least one adverse obstetric outcome. Accordingly, the admission status of mothers, residence, ANC follow-up, the onset of labor, iron foliate supplementation, gestational age at delivery, and hemoglobin level were significant at *p* < 0.25. After controlling for confounding variables, the odds of adverse obstetric outcomes among women with preeclampsia were 2.25 times higher than those of normotensive women (adjusted odds ratio [AOR: 2.25, 95% CI: (1.06, 4.77)]) (Table 4).

**Table 4:** Bivariate and multivariate regression analyses of maternal preeclampsia, adverse obstetrical, and perinatal outcomes among women who gave birth at WCSH, Northeast Ethiopia, 2023.

Outcome variable		Women with preeclampsia	Normotensive women	Crude OR (95% CI)	Adjusted OR (95% CI)	P-value
^1^Adverse obstetrical outcomes	Yes No	31 109	19 261	3.91 (2.12, 7.21) 1	2.25 (1.06, 4.77)^*^ 1	0.03
^2^Low birth weight	Yes No	44 96	20 260	5.96 (3.34, 10.62) 1	3.11 (1.43, 6.76) ^*^ 1	0.00
^3^Preterm birth	Yes No	42 98	17 263	6.63 (3.61, 12.19) 1	2.21 (1.02, 4.81) * 1	0.04
^4^Perinatal Asphyxia	Yes No	26 114	16 264	3.76 (1.94, 7.28) 1	2.53 (1.15, 5.54) *	0.02
^5^Perinatal death	Yes No	23 117	8 272	6.68 (2.91,15.38) 1	2.90 (1.10,8.17)* 1	0.04
^6^5^th^ minute Low Apgar	Yes No	17 101	16 259	0.37 (0.18–0.75) 1	0.62 (0.26–1.50) 1	0.29

^*^p < 0.05, 1 = reference (normotensive women), OR = odds ratio.

^1^Adjusted for admission status of mothers, residence, ANC follow-up, onset of labor, iron foliate supplementation, gestational age at delivery, and hemoglobin level in mg/dl.

^2^Adjusted for admission status of mothers, ANC follow-up, onset of labor, gestational age, and Hgb level in mg/dl.

^3^Adjusted for admission status of mothers, number of ANC visits, and onset of labor.

^4^Adjusted for admission status of mothers, onset of delivery, mode of labor, iron-folic acid supplementation, and parity.

^5^Adjusted for admission status of mothers, gestational age, iron-folic acid supplementation, onset of labor, and mode of delivery.

^6^Adjusted for mode of delivery, onset of labor, admission status of mothers, number of ANC visits, and gestational age in weeks.

Furthermore, this study revealed that women with preeclampsia had 2.90 times higher odds of perinatal death compared to those without preeclampsia [AOR: 2.90; 95% CI: 1.10, 8.17]. In addition, babies born to women with preeclampsia were 3.11 times more likely to have low birth weight [AOR: 3.11; 95% CI: 1.43, 6.7] and 2.53 times more likely to have birth asphyxia [AOR: 2.53, 95% CI: 1.15, 5.5] than babies born to normotensive women (Table 4).

## Discussion

Preeclampsia poses a substantial threat to maternal and perinatal health, contributing significantly to adverse outcomes for both mothers and their infants, particularly in developing nations such as Ethiopia (27). This study also highlights the vulnerability of women with preeclampsia to adverse obstetric and perinatal outcomes compared to normotensive women. The presence of preeclampsia was associated with an increased likelihood of experiencing at least one adverse obstetric outcome. Furthermore, neonates born to preeclamptic women had a higher likelihood of perinatal death, low birth weight, birth asphyxia, and preterm birth than those born to normotensive women.

This study revealed a higher proportion of antepartum hemorrhage, postpartum hemorrhage, and blood transfusions in women with preeclampsia compared to those who were normotensive. Similar findings were observed across diverse geographic settings, including Brazil (28), Kenya (28), Ethiopia (17), and India (29). Similarly, the odds of experiencing at least one adverse obstetric outcome among women with preeclampsia were higher than those among normotensive women. These findings align with previous studies conducted in Ethiopia (19, 21). However, the proportions of these complications varied across the studies. This variation could potentially be attributed to differences in study design. Additionally, variations could be attributed to differences in the guidelines and criteria used for diagnosing postpartum hemorrhage or determining the need for blood transfusions in different healthcare settings. Collectively, these findings paint a concerning picture of the substantial maternal health challenges posed by preeclampsia.

Furthermore, the study revealed that the prevalence of perinatal death, low birth weight, preterm birth, and composite adverse perinatal outcomes was higher in the preeclamptic group than in the normotensive group. This finding is consistent with previous studies conducted in Ethiopia (19, 20, 26, 30, 31), Nigeria (32), India (33, 34), Haiti (35), Bahrain (36), and Ghana (37). The observed neonatal complications may be attributed to the increased emphasis on improving newborn and perinatal care, which aligns with the global efforts to decrease neonatal mortality and morbidity and achieve the Sustainable Development Goals at both the national and international levels. Specific interventions implemented to enhance newborn care including the establishment or strengthening of neonatal intensive care units are vital.

Newborns born to preeclamptic women were found to have a higher likelihood of having low birth weight than those born to normotensive women. This finding aligns with a study conducted in southern Ethiopia (31). Furthermore, this study revealed that neonates born to preeclamptic women had a significantly higher risk of perinatal death, preterm birth, and perinatal asphyxia. Preeclampsia is associated with impaired fetal growth, resulting in low birth weight and related complications (38). Low birth weight is a significant consequence of preeclampsia due to fetal poor nutrition caused by inadequate uteroplacental vascular function. This condition leads to growth retardation and increases the risk of fetal morbidity including premature birth and mortality (39, 40). The consistent evidence from diverse settings emphasizes the urgent need to prioritize the prevention, early identification, and comprehensive management of preeclampsia as a crucial aspect of maternal and newborn health.

## Conclusion and recommendations

In this study, adverse obstetric and perinatal outcomes were observed more commonly in women with preeclampsia than in normotensive women. Perinatal death, low birth weight, asphyxia, and preterm birth were significantly associated with preeclampsia. To achieve the Sustainable Development Goals, it is crucial for governments to prioritize the reduction of adverse outcomes related to preeclampsia in mothers and newborns. Healthcare providers play a vital role in promoting early detection, prompt intervention, and prevention of preeclampsia. Additionally, the implementation of calcium supplementation programs should be prioritized, especially in regions with inadequate dietary intake of calcium, as it has been shown to reduce the risk of preeclampsia (41). Furthermore, it is essential to conduct a prospective cohort study to evaluate the impact of various types of hypertensive disorders on adverse maternal and perinatal outcomes during pregnancy.

## Limitation of the study

The primary limitation of this study is that it was a single-center study, which may limit the generalizability of the findings to other healthcare settings. Furthermore, this study included only women who attended health institutions. Therefore, the results of this study may not represent the obstetric and perinatal outcomes of women with and without preeclampsia in the community in countries with poor healthcare-seeking behavior, such as Ethiopia.

## Ethical considerations

Ethical clearance for the study was obtained from the Institutional Review Board (IRB) of Woldia University. The specific protocol number was WDU/IRB001, and the approval was dated March 06, 2023. However, because the study was conducted by reviewing medical records, confidentiality and anonymity were ensured throughout the study. Further permission for the utilization of charts was obtained from the medical director of the WCSH and the department head of the maternity ward.

## Data Availability

The raw data supporting the conclusions of this article will be made available by the authors, without undue reservation.
